# Generation of bioartificial hearts using decellularized scaffolds and mixed cells

**DOI:** 10.1186/s12938-019-0691-9

**Published:** 2019-06-04

**Authors:** Cailing Tong, Cheng Li, Baiyi Xie, Minghui Li, Xianguo Li, Zhongquan Qi, Junjie Xia

**Affiliations:** 1 School of Life Science, Xiamen University, Xiamen, 361102 Fujian China; 2 Organ Transplantation Institute of Xiamen University, Fujian Provincial Key Laboratory of Organ and Tissue Regeneration, School of Medicine, Xiamen University, Xiamen, 361102 Fujian China; 3 School of Medicine, Guangxi University, Nanning, 530004 Guangxi China

**Keywords:** Bioartificial heart, Decelluarized scaffold, Mesenchymal stem cells, Myocardial cells, Endothelial cells

## Abstract

**Background:**

Patients with end-stage heart failure must receive treatment to recover cardiac function, and the current primary therapy, heart transplantation, is plagued by the limited supply of donor hearts. Bioengineered artificial hearts generated by seeding of cells on decellularized scaffolds have been suggested as an alternative source for transplantation. This study aimed to develop a tissue-engineered heart with lower immunogenicity and functional similarity to a physiological heart that can be used for heart transplantation.

**Materials and methods:**

We used sodium dodecyl sulfate (SDS) to decellularize cardiac tissue to obtain a decellularized scaffold. Mesenchymal stem cells (MSCs) were isolated from rat bone marrow and identified by flow cytometric labeling of their surface markers. At the same time, the multi-directional differentiation of MSCs was analyzed. The MSCs, endothelial cells, and cardiomyocytes were allowed to adhere to the decellularized scaffold during perfusion, and the function of tissue-engineered heart was analyzed by immunohistochemistry and electrocardiogram.

**Results:**

MSCs, isolated from rats differentiated into cardiomyocytes, were seeded along with primary rat cardiomyocytes and endothelial cells onto decellularized rat heart scaffolds. We first confirmed the pluripotency of the MSCs, performed immunostaining against cardiac markers expressed by MSC-derived cardiomyocytes, and completed surface antigen profiling of MSC-derived endothelial cells. After cell seeding and culture, we analyzed the performance of the bioartificial heart by electrocardiography but found that the bioartificial heart exhibited abnormal electrical activity. The results indicated that the tissue-engineered heart lacked some cells necessary for the conduction of electrical current, causing deficient conduction function compared to the normal heart.

**Conclusion:**

Our study suggests that MSCs derived from rats may be useful in the generation of a bioartificial heart, although technical challenges remain with regard to generating a fully functional bioartificial heart.

## Background

Currently, heart transplantation remains the final treatment for end-stage heart failure patients [1], and it is estimated that thousands of end-stage heart failure patients wait for heart transplantations annually worldwide [2], while the supply of donor hearts is limited [3]. Although many measures have been taken to increase the source of organs, including the use of xenografts [4–6] and the development of methods for organ preservation [7], chimera technology [8, 9], and in vitro organ culture [10], the situation has not been significantly improved. Although the use of animal organs/tissues involves some ethical issues [11], it does bring new hope to the field of organ transplantation [12]. For instance, xenografts from animal donors such as the chimpanzee and baboon greatly expand the source of donor organs [13]. However, due to issues such as immune rejection and the potential spread of animal diseases to humans, the development of clinical approaches to using animal organs/tissues in humans has been limited [14–16]. Patients who accept xenograft hearts require lifelong immunosuppression, which decreases their quality of life [17, 18]. In recent decades, researchers have proposed the use of an acellular scaffold to generate functional hearts in vitro for transplantation. Several systems have been reported, including a technique for in vitro cultured heart, which unfortunately showed signal disorder and negatively affected cardiac function [10]. The field of tissue-engineering seeks to reconstitute functional organs including the heart using various cell types and scaffold materials. Biomaterials derived from cells onto decellularized scaffolds have been proposed as an alternative approach to bioengineering artificial hearts for transplantation [19]. When a decellularized scaffold is derived from an organ of an animal, it offers a three-dimensional scaffold structure that is non-cytotoxic, promotes cell adhesion and proliferation, and represents an ideal material for tissue engineering [20]. Thus far, decellularized scaffolds have been used in an effort to construct organs including heart [21], and a bioartificial heart with low potential for immunological rejection represents a promising approach to solve the problem of donor heart deficiency.

With the development of stem cell technology, the use of such cell types to generate a functional bioartificial heart has gained increasing attention in recent years. Cardiomyocytes differentiated from embryonic stem cells [22], inducible pluripotent stem cells (iPSCs) [23], and human mesenchymal stem cells (MSCs) [24] have been utilized in an effort to generate bioartificial hearts. In particular, MSCs are multipotent stem cells present in adult tissues that possess the ability to differentiate into a variety of tissues and have been applied in the treatment of heart disease [25]. Moreover, MSCs exhibit low immunogenicity [26]. Hence, MSCs are an ideal cell type for seeding onto scaffolds for the purpose of generating a bioartificial heart. In the present study, we isolated MSCs from rats and confirmed their pluripotency. We then used these rat MSCs together with isolated rat cardiac and endothelial cells to generate bioartificial hearts on decellularized rat heart scaffolds.

## Materials and methods

### Animals

Adult male Lewis rats (4- to 6-week old, weighing 150–180 g, healthy) and neonatal Lewis rats (1- to 3-day old, healthy) were purchased from SLAC Laboratory Animal Co. The animals were housed at the Xiamen University Animal Center. All animals were anesthetized prior to harvesting of hearts and were humanely euthanized after the procedures were completed. The animal experiments were approved by the Animal Center of Xiamen University (Xiamen, China) and complied with the Instructive Notions with Respect to Caring for Laboratory Animals, 2006, published in by the Science and Technology Department of China.

### Isolation of rat MSCs

Adult Lewis rats were anesthetized with an intraperitoneal injection of chloralic hydras (3.8 mg/kg), sterilized with 70% ethanol, and skin and muscle were removed from the limbs. The marrow was removed by rinsing the marrow cavity repeatedly with phosphate-buffered saline (PBS). Broken bones were collected with sterile hemostatic forceps and digested with 1 mg/mL collagenase II in a 37 °C shaker bath for 1 h. After removal of the collagenase II solution, the digested cells were cultured in Dulbecco’s Modified Eagle Medium (DMEM, Gibco) with 10% fetal bovine serum (FBS), 1× non-essential amino acids (NEAA, Millipore), 1× 2-mercaptoethanol (Gibco), 10 ng/mL fetal growth factors (FGFs, Peprotech), and 10 ng/mL epidermal growth factor (EGF, Peprotech).

### Cell-surface antigen profiling of MSCs

The cell-surface antigen profile of rat MSCs was analyzed as previously described [27, 28]. The isolated MSCs were incubated with fluorescein isothiocyanate (FITC)-conjugated antibodies against CD106, CD25, CD44, CD45, CD11b, CD29, and CD90, and phycoerythrin (PE)-conjugated antibodies against CD34 and CD71. All incubations were performed at 4 °C for 30 min. Quantitative analyses were performed using a Beckman Coulter flow cytometer (Beckman Coulter).

### Multi-directional differentiation of rat MSCs

A MSC differentiation kit (Cyagen) was used to direct the differentiation of MSCs into osteoblasts, adipogenic cells, and chondroblasts according to the manufacturer’s instructions.

### Lentivirus generation and infection

Lentivirus packaging was performed as previously described with some modifications [29, 30]. The pLL3.7 empty vector and Pmd2G, pRSV-Rev, VSVG helper vectors were used for lentivirus packaging in 293T cells. Plasmids were prepared with the EndoFree system (Qiagen) according to the manufacturer’s instruction. The packaged virions that contained green fluorescent protein (GFP) were concentrated for infection of isolated MSCs. MSCs with lentiviral-aided expression of GFP were sorted by flow cytometry (MoFlo XDP, Beckman).

### Isolation of neonatal rat cardiomyocytes

Rat neonatal cardiomyocytes were isolated as previously described [31]. In brief, neonatal rats were anesthetized with 5% inhaled isoflurane, and the chest was sterilized with 70% ethanol. The hearts were excised, minced, and incubated with 0.2% collagenase II solution (Beijing Solarbio Science & Technology Co., Ltd.,) in a 37 °C shaker bath for 45 min. The digested cells were filtered through a 40- to 50-μm nylon mesh and collected by centrifugation at 1000 rpm for 5 min. The collected cells were pre-plated in the cardiomyocyte medium for 30 min, and the supernatant containing cardiomyocytes was collected and cultured in the presence of 0.1 mmol/L 5-BrdU for 48 h. The cardiomyocytes were then ready for subsequent experiments.

The cardiomyocyte medium consisted of DMEM (Gibco) supplemented with 10% FBS (BI), 5% horse serum (Beijing Solarbio Science & Technology Co., Ltd.), 100 U/mL penicillin-G (Gibco), 100 U/mL streptomycin (Gibco), 0.05 mmol/L 2-mercaptoethanol (Gibco), 1.2 mM CaCl (Fisher), 0.8 mM MgCl (Sigma), and 10 nM isoprenaline. The cardiomyocyte medium was used in all ECM recellularization experiments involving neonatal cardiomyocytes.

### Characterization of MSC-derived cardiomyocytes

Induction of MSC differentiation into cardiomyocytes was performed as previously described with slight modifications [27]. In brief, GFP-expressing MSCs were co-cultured with isolated neonatal rat cardiomyocytes at a ratio of 5000:5000 cells on a slide for 1 week, followed by immunofluorescence analysis. The medium was replenished every 3 days.

Differentiated MSCs were characterized by immunocytochemistry analysis as previously described [32]. In brief, cells were fixed in 4% paraformaldehyde for 10 min, permeabilized with 0.5% Triton X-100 for 20 min, and blocked in 10% donkey serum (Beijing Solarbio Science & Technology Co., Ltd.). Cells then were incubated with antibodies against cTnT (1:400, Abcam) and desmin (1:100, Abcam) at 4 °C overnight. The cells were then further incubated with the appropriate secondary antibodies (Abcam) diluted at 1:100 for 1 h at room temperature, followed by another incubation with DAPI (Beijing Solarbio Science & Technology Co., Ltd.). Untreated rat MSCs and adult cardiomyocytes were used as negative and positive controls, respectively. The results of immunostaining were observed under fluorescence microscopy (Olympus FV1000).

### Isolation of rat endothelial cells

Adult Lewis rats were anesthetized as described above. Each aorta was collected, placed immediately into a 10-cm diameter dish containing 7 mL D-Hank’s buffer (Beijing Solarbio Science & Technology Co., Ltd.), and washed with cold D-Hank’s buffer by pipetting. The connective tissues were removed, and the remaining tissues were minced into pieces smaller than 1 mm^3^. These tissues were then digested in 10 mg/mL collagenase II (Gibco) at 37 °C for 15 min. The isolated endothelial cells were collected by centrifugation and then cultured in 10-cm diameter culture dishes pre-coated with 1% collagen in high-glucose DMEM containing 10% FBS, 50 U/mL heparin, 100 U/mL penicillin-G (Gibco), and 100 U/mL streptomycin (Gibco).

### Determination of CD31 expression on rat endothelial cell

Rat endothelial cells were trypsinized (0.25% trypsin, Gibco), collected, and stained with a monoclonal antibody against PeCy7-CD31 (Thermo Fisher Scientific) according to the manufacturer’s recommendations. Incubations were performed at 4 °C for 30 min. Quantitative analyses were performed using a Beckman Coulter flow cytometer (Beckman Coulter).

### Characterization of MSC-derived endothelial cells

MSCs and endothelial cells were co-cultured at a ratio of 200,000:200,000 cells in 10-cm dishes in the endothelial cell medium at 37 °C in a humidified atmosphere containing 5% CO_2_. The medium was replenished every 3 days. After 1 week in co-culture, the cells were subjected to flow cytometric analysis as described above.

### Decellularization of rat hearts

Decellularized cardiac scaffolds were produced as described previously with some modifications [31]. In brief, the heart was removed from the anesthetized rat and perfused through the ascending aorta for 3 days with 200 mL of PBS containing heparin (20 U/mL) and 10 mM adenosine followed by 2 L of 0.1% sodium dodecyl sulfate, 200 mL of deionized water, 200 mL of 1% Triton X-100, and finally, 2 L of PBS containing 100 U/mL penicillin-G (Gibco), 100 U/mL streptomycin (Gibco), and 100 U/mL amphotericin B (Sigma-Aldrich). A total of 7 rat hearts were used in this study.

### Recellularization of decellularized rat hearts

To generate a bioartificial heart on a decellularized cardiac scaffold, we first decellularized rat hearts to prepare a scaffold and then reintroduced cells within the scaffold by seeding MSCs along with primary cardiomyocytes and endothelial cells. We mounted each recellularized whole rat heart in a bioreactor that provided coronary perfusion with oxygenated culture medium for 10 days and then analyzed the condition of the adhered cells by immunocytochemical staining. In brief, for the first 3 days of perfusion described above, we continued the perfusion of the cardiac scaffolds with cardiomyocyte medium and injected 1 mL medium containing 1 × 10^7^ cardiomyocytes, 1 × 10^6^ endothelial cells, and 5 × 10^7^ MSCs into the decellularized heart through the aortic arch. Perfusion was discontinued for 4 h to allow cell adhesion and then resumed with 37 °C oxygenated cardiomyocyte medium for 10 days.

### Electrocardiography (ECG)

Electrocardiography was performed on the bioartificial heart using the RM6240E multi-channel physiological signal acquisition processing system (Chengdu Instrument Factory) on day 10 after cell seeding. The bioartificial heart was removed from the bioreaction system and fixed onto the RM6240E apparatus for ECG recording with a voltage of 2–20 Mv, a delay of 2 s, and a continuous single stimulus.

### Immunohistochemistry analysis of the bioartificial heart

The bioartificial heart was removed from the bioreaction system, washed twice with PBS, fixed in 4% paraformaldehyde (Beijing Solarbio Science & Technology Co., Ltd.), and embedded in paraffin. Bioartificial heart was then cut and sectioned into 5-µm-thick sections. The sections were used for immunohistochemical staining following standard protocols. In brief, the sections were incubated with primary antibodies against cTnT and desmin, followed by another incubation with the appropriate secondary antibodies and DAPI. Fluorescence signals were captured under a fluorescence microscope (Olympus FV1000).

## Results

### Isolation, characterization, and multipotent differentiation of rat MSCs

**Fig. 1:**
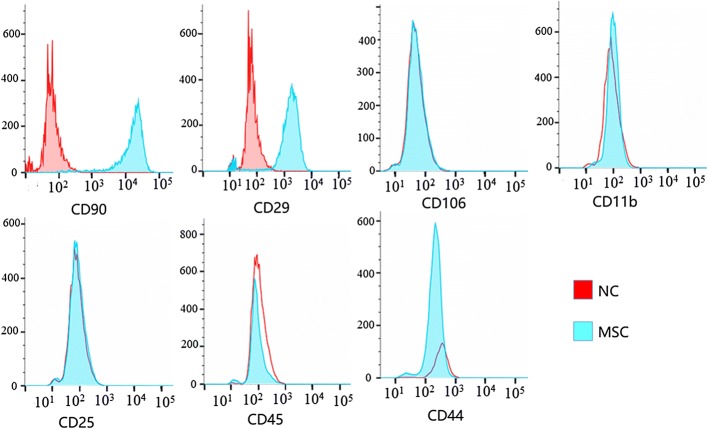
Flow cytometric determination of cell-surface antigen profile of rat MSCs. Rat MSCs were stained with specific antibodies against a variety of cell-surface antigens as indicated. The normal control (NC) samples are MSCs without cell-surface staining. The percentage of cells that stained positively for each antibody is listed in the respective histogram. Similar results were obtained from MSCs isolated from two separate rats. Representative flow cytometric images from one rat are shown

**Fig. 2:**
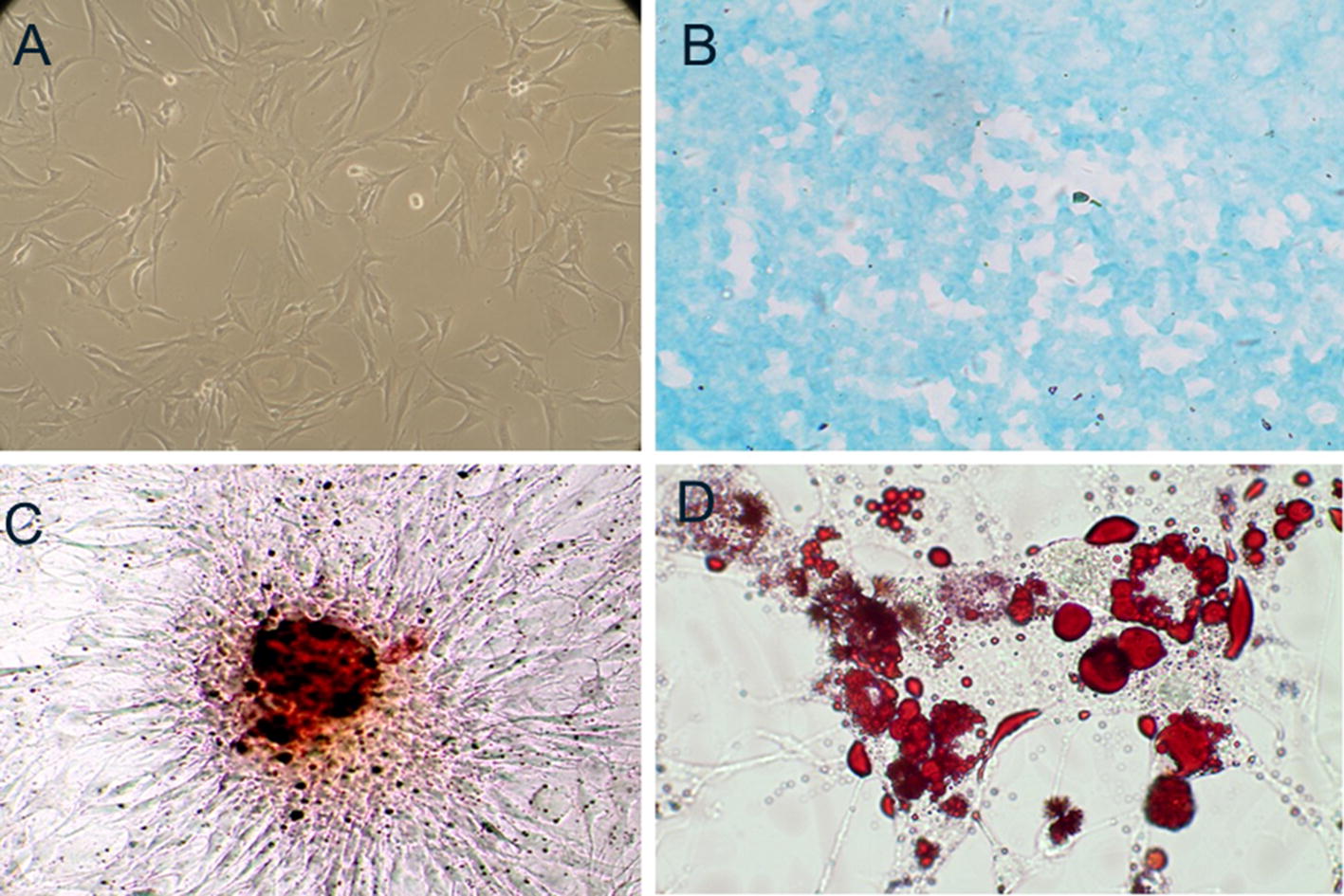
Rat MSCs and their multilineage differentiation potential. **A** The appearance and growth of MSCs of the third passage. **B** Chondroblast differentiation was demonstrated by Alcian blue staining. **C** Osteogenic differentiation was demonstrated by alizarin red staining. **D** Adipogenic differentiation was demonstrated by Oil Red O staining

We first characterized the molecular properties of the rat MSCs isolated for this study by flow cytometry. As shown in Fig. 1, the rat MSCs expressed the MSC surface markers CD90 and CD29, but were negative for markers typically absent on MSCs, including CD44, CD106, CD25, CD11b, and CD45. Also, rat MSCs exhibited a spindle-shaped morphology (Fig. 2A), further confirming that the isolated rat MSCs in the present study displayed the morphological and molecular characteristics typical of MSCs.

To confirm the differentiation potential of rat MSCs, MSCs at the third passage were induced to differentiate into adipocytes, osteoblasts, and chondroblasts, separately. After 30 days of culture in chondroblast induction medium, the cells exhibited a rounded cell body and expressed acid mucopolysaccharides as demonstrated by Alcian blue staining (Fig. 2B). After 20 days in osteogenic induction medium, most of the cells were positive for alkaline phosphatase expression and had formed aggregates or nodules with calcium mineralization as observed by alizarin red staining (Fig. 2C). Adipogenic differentiation was induced by 1 week of culture in adipogenic induction medium, and by 3 weeks later, almost all cells contained Oil Red O-stained lipid droplets (Fig. 2D). Hence, we concluded that the isolated rat MSCs exhibited the capacity for multipotent differentiation.

### MSC differentiation into cardiomyocytes or endothelial cells

**Fig. 3:**
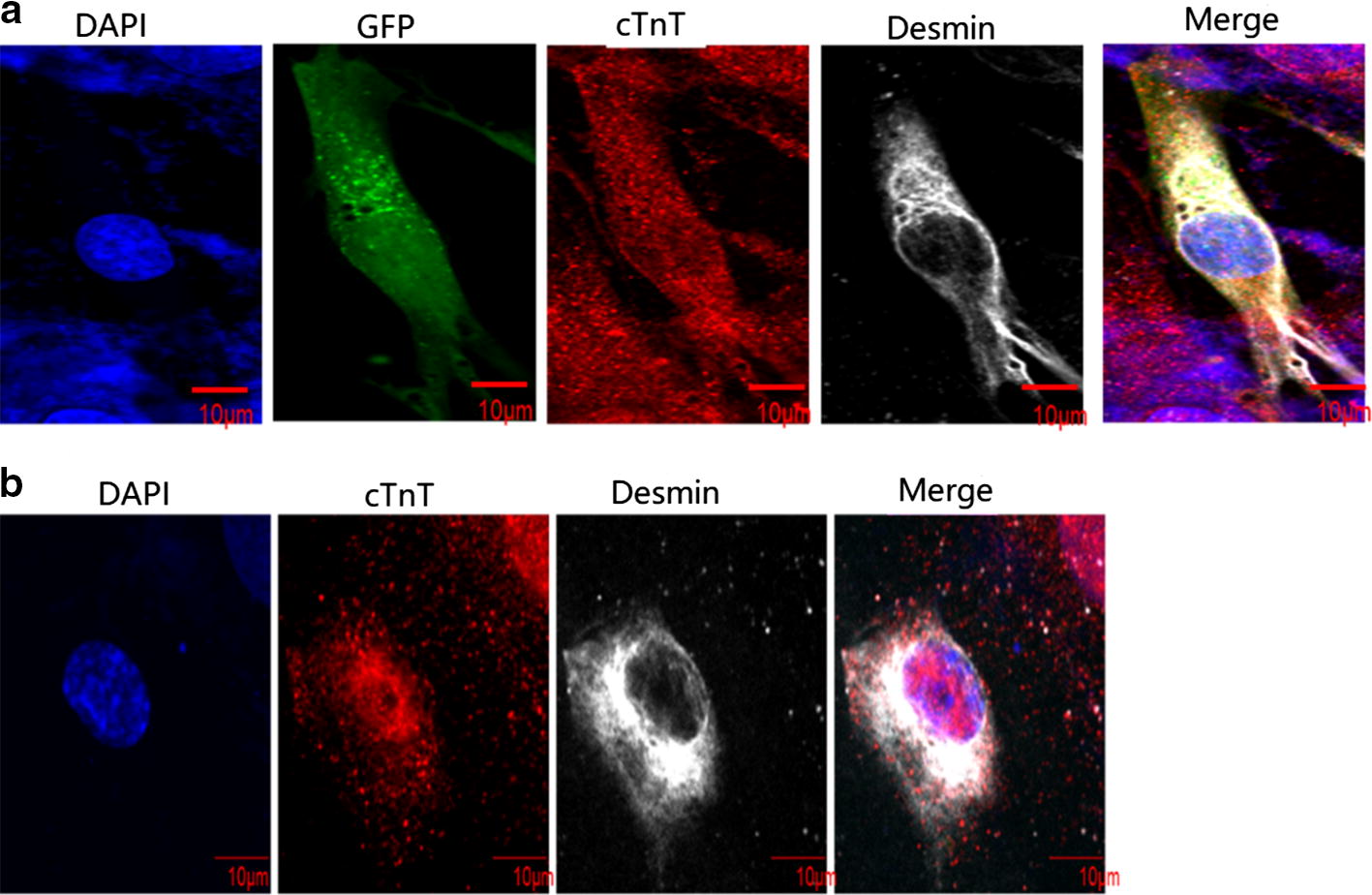
Differentiation of rat MSCs into cardiomyocyte. **a** GFP-expressing MSCs were co-cultured with isolated newborn rat cardiomyocytes. **b** Newborn rat cardiomyocytes. Cells were stained for desmin (silvery white), cTnT (red), and DAPI (blue, nuclear staining)

We next examined the capability of the isolated MSCs to differentiate into cardiomyocytes. After co-culture with isolated newborn rat cardiomyocytes, we found that rat MSCs exhibited positive staining for desmin and cTnT, two widely used cardiomyocyte markers (Fig. 3a), at levels similar to those in the newborn rat cardiomyocytes (Fig. 3b).

**Fig. 4:**
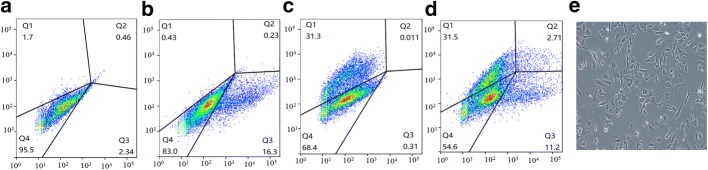
Differentiation of rat MSCs into endothelial cells. **a** Rat endothelial cells served as a positive control. **b** GFP-expressing MSCs co-cultured rat endothelial cells. **c** Rat endothelial cells stained with antibody against CD31. **d** MSC-derived endothelial cells were stained with the antibody against CD31. **e** Morphology of rat endothelial cells

We also examined the capability of MSCs to differentiate into endothelial cells. After co-culture with isolated rat endothelial cells, the isolated rat MSCs stained positively for CD31. Untreated rat MSCs were used as negative controls, and co-cultured MSCs showed low expression of CD31 (Fig. 4).

### Formation of bioartificial heart on decellularized scaffold

**Fig. 5:**
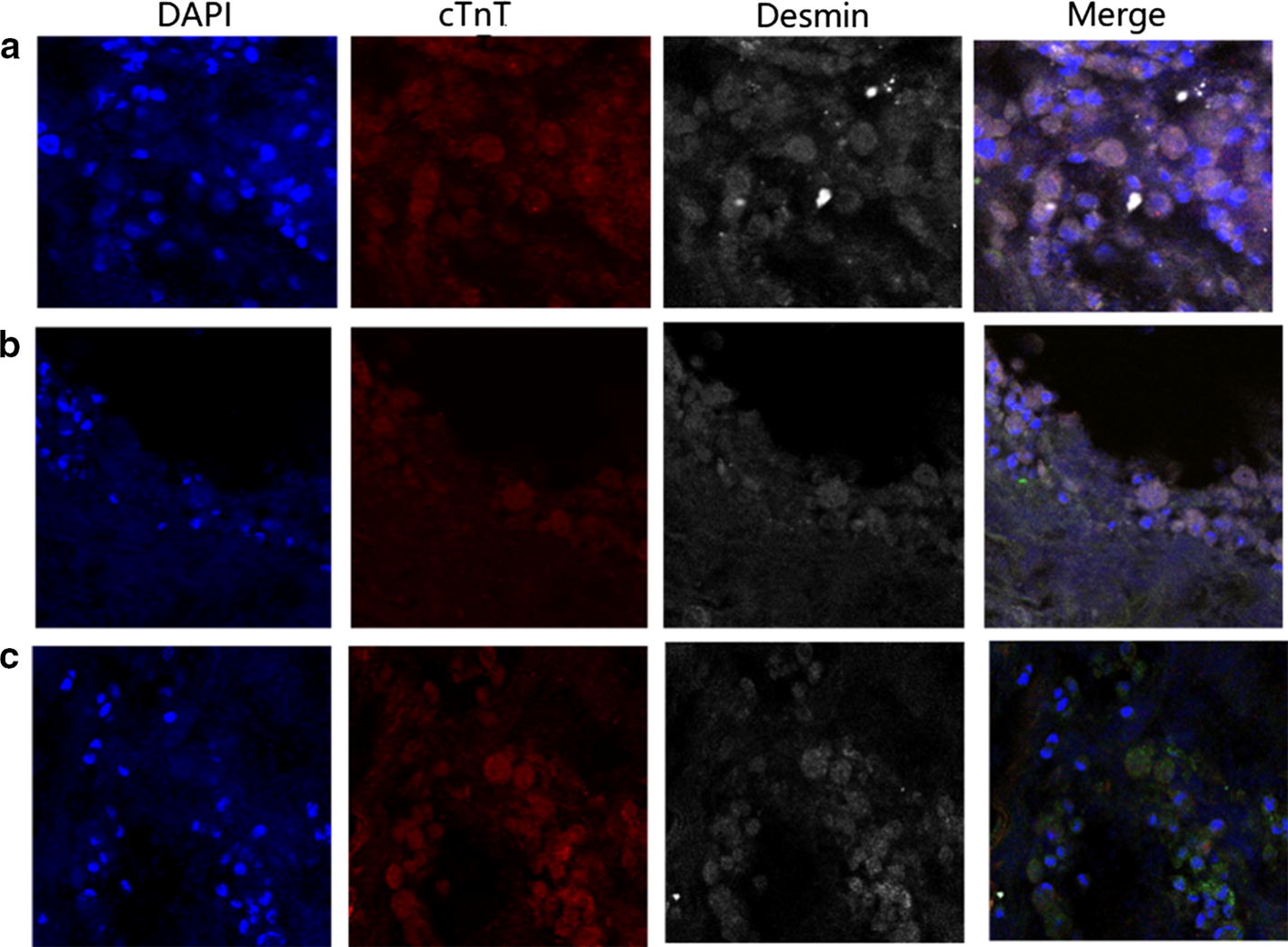
Histological analysis of engineered heart tissues. **a** Cardiac wall, **b** artery, and **c** cardiac chamber of bioartificial heart. **a**–**c** Immunostained with antibodies against cTnT and desmin. DAPI was used for nuclear counterstaining

**Fig. 6:**
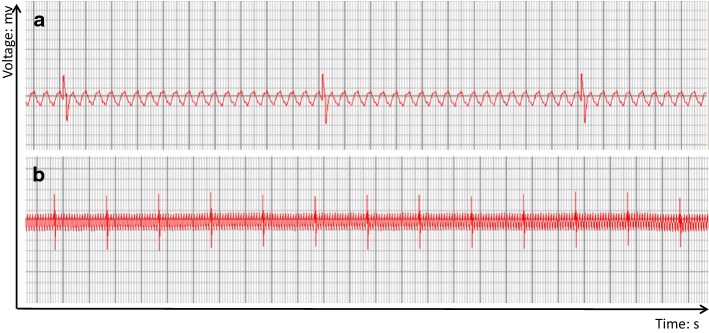
ECG of bioartificial heart. Representative images from the electrical activity assessment of the bioartificial whole heart after 10 days in a heart bioreactor. **a** Part of Magnified electrocardiogram of bioartificial heart. **b** Part of electrocardiogram of bioartificial. The abscissa is time, the smallest grid is 0.04 s, and the big grid is 0.2 s. The ordinate is mv, with 0.1 mv per small cell, thus, five small cells (one large cell) is 0.5 mv, and the length of two large cells is 1 mm

**Fig. 7:**
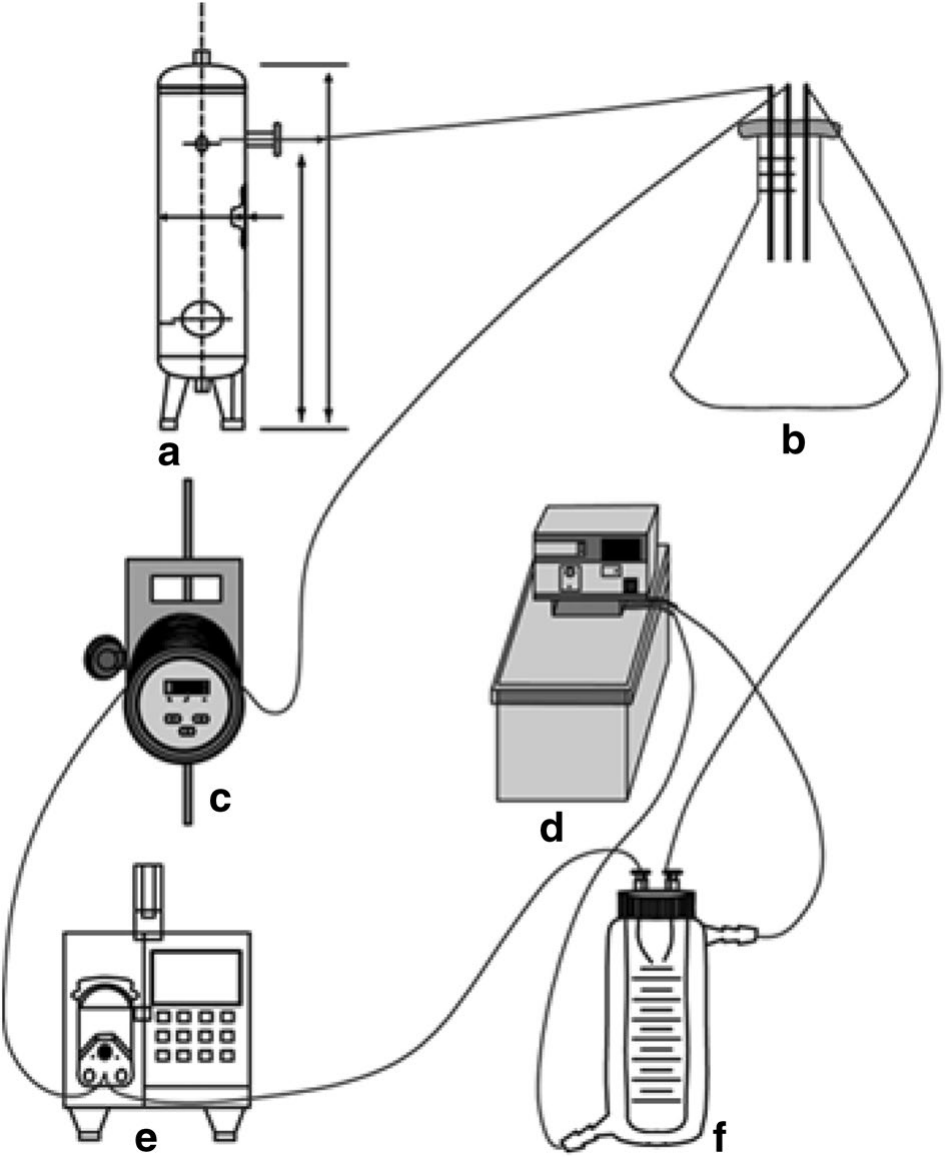
Diagram showing the in vitro culture system for the bioartificial heart. **a** Mixed gas tank used for gas supply in organ culture. The main component is a mixture of oxygen and carbon dioxide. The amount of the mixed gas may be adjusted by the valve to ensure the percentage of oxygen in the mixed air, so that the medium supply ensures that the organ receives sufficient amount of oxygen during the cultivation process. **b** Three-hole glass bottle. The three holes are connected to the mixed gas, the inlet, and the outlet of the organ culture bottle through a hose. The medium containing saturated gas can be recycled through this bottle. **c** Disposable infusion tube heater. This heater can heat the culture medium pumped from the three-hole glass bottle at a constant temperature so that the medium entering the organ is maintained at an optimum temperature of 37 °C. **d** Thermostatic bath. This device has both heating and pumping functions, which can maintain the outer tube of the F device at 37 °C water circulation, thereby providing the optimum ambient temperature for the organ. **e** Titration pump. This device can continuously and quantitatively draw the culture medium from B into F, thereby ensuring that the organ is cultured in a nutrient-rich fresh medium. **f** Organ culture bottle. This device is divided into an outer tube and an inner tube, and these two are not connected. The outer tube is maintained at a constant temperature of 37 °C by connecting to a constant temperature bath. The organ is fixed by two hollow tubes of the cap and placed in the inner tube for cultivation. The entire system can be sealed to ensure a sterile environment

We culture bioartificial heart at 37 °C in a humidified atmosphere containing 5% CO_2_. We found that the cells adhered to the cardiac wall, artery, and cardiac chamber of the decellularized scaffold stained positively for cTnT and desmin (Fig. 5). Also, the bioartificial heart was able to generate a certain level of electrical activity as detected on ECG, but displayed only *R* wave with no *P* wave (Fig. 6). The in vitro culture system for bioartificial heart is shown in Fig. 7.

## Discussion

In the present study, we generated bioartificial hearts with rat MSCs, endothelial cells, and myocardial cells seeding onto decellularized rat heart scaffolds. However, our bioartificial hearts possessed only partial electrical activity as evaluated by ECG.

With continued advances in our understanding of stem cell biology and iPSCs as well as related technologies, the generation of bioartificial hearts has been an active area of research in recent decades. However, clinical research has yet to produce a viable treatment using this technology [33], suggesting the persistence of obstacles for the use of bioartificial hearts in human patients. To date, several approaches to generating bioartificial hearts have been reported, including the use of iPSCs [33] and three-dimensional printing [34, 35]. However, the safety and immunogenicity of embryonic stem cells and iPSCs are major concerns [36]. Compared to synthetic chemical materials, decellularized scaffolds are derived from physiological materials, are non-cytotoxic, and promote cell adhesion [37]. In the present study, we employed a decellularized rat heart as a scaffold, which is an established method for preparing a foundation for the basic cardiac structure [38]. We recellularized these scaffolds with MSCs together with endothelial and cardiac cells isolated from rats. We first characterized the multipotency of the isolated MSCs, including their differentiation into cardiomyocytes by immunostaining against the specific cardiac markers cTnT and desmin as well as into endothelial cells according to expression of the marker CD31. We then showed that these cells would adhere and differentiate on decellularized rat heart scaffolds.

While we successfully recellularized rat heart scaffolds with cardiac cells toward the generation of bioartificial heart, unfortunately, it only exhibited partial electrical function on ECG. We found that the bioartificial heart only displayed *R* wave without *P* wave, indicating that ventricular electrical conduction was normal but atrial electrical conduction was abnormal. Thus, it appeared that our bioartificial heart lacked the necessary conducting cells, consistent with a previous report [39]. Compared with MSCs, induced pluripotent stem cells and embryonic stem cells can be more stably differentiated into cardiomyocytes, but there is no general method for their use in the construction of a functional tissue-engineered heart. A functional heart requires multiple cells, each of which needs to be re-differentiated [40]. Cardiomyocytes from induced pluripotent stem cells and embryonic stem cells have similar maturity to neonatal cardiomyocytes, but the method of inducing differentiation directly leads to electrophysiological changes [41]. Through differences in culture and differentiation conditions, these stem cells can be induced to differentiate into tissue-engineered heart cells that have electrophysiology, and electrical conduction [42]. However, it has also been reported that tissue-engineered hearts constructed using induced stem cells not only exhibit ECG properties close to those of the physiological heart, which has *P* waves and *R* waves, but also show a difference between waveform and interval time and normal [23, 31]. Therefore, determining how to generate a bioartificial heart functionally capable of physiological conduction remains a challenge in the field.

Several limitations of this study need to be acknowledged. First, while ECG recording was performed from the bioartificial hearts, we did not obtain ECG recordings from normal hearts from our rats for direct comparison. Also, the electrophysiological properties of the MSC-derived cardiomyocytes were not characterized at the single-cell level. We plan to characterize the electrical properties of these cells by patch-clamp recording in our future studies. In addition, we did not perform mechanical testing on the bioartificial hearts or measure blood flow through them due to equipment unavailability. Finally, we did not perform any statistical analysis of the results in this study.

## Conclusions

In conclusion, we demonstrated in the present study that MSCs isolated from rats may be used to generate bioartificial hearts together with isolated endothelial and cardiac cells on a decellularized heart scaffold. However, technical challenges remain with regard to the generation of a fully functional bioartificial heart to meet the pressing clinical need for donor hearts.

## Data Availability

Data sharing not applicable to this article as no datasets were generated or analyzed during the current study.

## Abbreviations

MSCs: mesenchymal stem cells
iPSCs: inducible pluripotent stem cells
DMEM: Dulbecco’s Modified Eagle Medium
FBS: fetal bovine serum
PE: phycoerythrin
GFP: green fluorescent protein
cTnT: cardiac troponin T
ECG: electrocardiogram
PBS: phosphate-buffered saline
FITC: fluorescein isothiocyanate
DAPI: 4′,6-diamidino-2-phenylindole, dihydrochloride
SDS: sodium dodecyl sulfate
FGFs: fetal growth factors
EGF: epidermal growth factor

## References

[1] Katz JN, Waters SB, Hollis IB, Chang PP (2015). Advanced therapies for end-stage heart failure. Curr Cardiol Rev.

[2] Mozaffarian D, Benjamin EJ, Go AS, Arnett DK, Blaha MJ, Cushman M (2015). Heart disease and stroke statistics—2015 update: a report from the American Heart Association. Circulation..

[3] Beyar R (2011). Challenges in organ transplantation. Rambam Maimonides Med J..

[4] Ekser B, Cooper DKC, Tector AJ (2015). The need for xenotransplantation as a source of organs and cells for clinical transplantation. Int J Surg.

[5] Dooldeniya MD, Warrens AN (2003). Xenotransplantation: where are we today?. J R Soc Med.

[6] Boksa M, Zeyland J, Słomski R, Lipiński D (2015). Immune modulation in xenotransplantation. Arch Immunol Ther Exp (Warsz).

[7] Giwa S, Lewis JK, Alvarez L, Langer R, Roth AE, Church GM (2017). The promise of organ and tissue preservation to transform medicine. Nat Biotechnol.

[8] Eckardt S, McLaughlin KJ, Willenbring H (2011). Mouse chimeras as a system to investigate development, cell and tissue function, disease mechanisms and organ regeneration. Cell Cycle.

[9] De Los Angeles A, Pho N, Redmond DE (2018). Generating human organs via interspecies chimera formation: advances and barriers. Yale J Biol Med.

[10] Al-Lamki RS, Bradley JR, Pober JS (2017). Human organ culture: updating the approach to bridge the gap from in vitro to in vivo in inflammation, cancer, and stem cell biology. Front Med.

[11] Bourret R, Martinez E, Vialla F, Giquel C, Thonnat-Marin A, De Vos J (2016). Human–animal chimeras: ethical issues about farming chimeric animals bearing human organs. Stem Cell Res Ther.

[12] Xue B, Li Y, He Y, Wei R, Sun R, Yin Z (2016). Porcine pluripotent stem cells derived from IVF embryos contribute to chimeric development in vivo. PLoS ONE.

[13] Starzl TE (1992). The future of xenotransplantation. Ann Surg.

[14] Michler Robert (1996). Xenotransplantation: Risks, Clinical Potential, and Future Prospects. Emerging Infectious Diseases.

[15] Joo HN, Kim S, Kang S, Lee KH (2010). The use of immunosuppressive drugs and legal implications in xenotransplantation. Mol Cell Toxicol.

[16] Starzl TE, Tzakis A, Fung JJ, Todo S, Demetris AJ, Manez R (1994). Prospects of clinical xenotransplantation. Transplant Proc.

[17] Aliabadi A, Cochrane AB, Zuckermann AO (2012). Current strategies and future trends in immunosuppression after heart transplantation. Curr Opin Organ Transplant.

[18] Page RL, Miller GG, Lindenfeld J. Drug therapy in the heart transplant recipient. Circulation. 2005. p. 111. http://circ.ahajournals.org/content/110/25/3858. Accessed 3 May 2017. 10.1161/01.CIR.0000151805.86933.35 15657387

[19] Yu Y, Alkhawaji A, Ding Y, Mei J (2016). Decellularized scaffolds in regenerative medicine. Oncotarget.

[20] Modulevsky DJ, Lefebvre C, Haase K, Al-Rekabi Z, Pelling AE (2014). Apple derived cellulose scaffolds for 3D mammalian cell culture. PLoS ONE.

[21] Moroni F, Mirabella T (2014). Decellularized matrices for cardiovascular tissue engineering. Am J Stem Cells.

[22] Vats A, Tolley NS, Bishop AE, Polak JM (2005). Embryonic stem cells and tissue engineering: delivering stem cells to the clinic. J R Soc Med.

[23] Lu T-Y, Lin B, Kim J, Sullivan M, Tobita K, Salama G (2013). Repopulation of decellularized mouse heart with human induced pluripotent stem cell-derived cardiovascular progenitor cells. Nat Commun.

[24] Toma C, Pittenger MF, Cahill KS, Byrne BJ, Kessler PD, Toma C (2002). Human mesenchymal stem cells differentiate to a cardiomyocyte phenotype in the adult murine heart. Circulation..

[25] Karantalis V, Hare JM (2015). Use of mesenchymal stem cells for therapy of cardiac disease. Circ Res..

[26] Gu L-H, Zhang T-T, Li Y, Yan H-J, Qi H, Li F-R (2015). Immunogenicity of allogeneic mesenchymal stem cells transplanted via different routes in diabetic rats. Cell Mol Immunol.

[27] Wang T, Xu Z, Jiang W, Ma A (2006). Cell-to-cell contact induces mesenchymal stem cell to differentiate into cardiomyocyte and smooth muscle cell. Int J Cardiol..

[28] Tsai MS, Lee JL, Chang YJ, Hwang SM (2004). Isolation of human multipotent mesenchymal stem cells from second-trimester amniotic fluid using a novel two-stage culture protocol. Hum Reprod..

[29] Stovall DB, Wan M, Zhang Q, Dubey P, Sui G (2012). DNA vector-based RNA interference to study gene function in cancer. J Vis Exp.

[30] Rubinson DA, Dillon CP, Kwiatkowski AV, Sievers C, Yang L, Kopinja J (2003). A lentivirus-based system to functionally silence genes in primary mammalian cells, stem cells and transgenic mice by RNA interference. Nat Genet.

[31] Ott HC, Matthiesen TS, Goh S-K, Black LD, Kren SM, Netoff TI (2008). Perfusion-decellularized matrix: using nature’s platform to engineer a bioartificial heart. Nat Med..

[32] Nethercott HE, Brick DJ, Schwartz PH (2011). Immunocytochemical analysis of human pluripotent stem cells. Methods Mol Biol.

[33] Gálvez-Montón C, Prat-Vidal C, Roura S, Soler-Botija C, Bayes-Genis A (2013). Cardiac tissue engineering and the bioartificial heart. Rev Española Cardiol.

[34] Ong CS, Yesantharao P, Huang CY, Mattson G, Boktor J, Fukunishi T (2018). 3D bioprinting using stem cells. Pediatr Res.

[35] Zhang YS, Yue K, Aleman J, Mollazadeh-Moghaddam K, Bakht SM, Yang J (2017). 3D bioprinting for tissue and organ fabrication. Ann Biomed Eng.

[36] Cao J, Li X, Lu X, Zhang C, Yu H, Zhao T (2014). Cells derived from iPSC can be immunogenic—yes or no?. Protein Cell.

[37] Cheng CW, Solorio LD, Alsberg E (2014). Decellularized tissue and cell-derived extracellular matrices as scaffolds for orthopaedic tissue engineering. Biotechnol Adv.

[38] Moser PT, Ott HC (2014). Recellularization of organs. Curr Opin Organ Transplant.

[39] Tao Z-W, Mohamed M, Hogan M, Salazar B, Patel NM, Birla RK (2015). Establishing the framework for fabrication of a bioartificial heart. ASAIO J..

[40] Liau B, Zhang D, Bursac N (2012). Functional cardiac tissue engineering. Regen Med.

[41] Lieu DK, Turnbull IC, Costa KD, Li RA (2012). Engineered human pluripotent stem cell-derived cardiac cells and tissues for electrophysiological studies. Drug Discov Today Dis Models.

[42] Eng G, Lee BW, Protas L, Gagliardi M, Brown K, Kass RS (2016). Autonomous beating rate adaptation in human stem cell-derived cardiomyocytes. Nat Commun.

